# The role of dual time point PET/CT for distinguishing malignant from benign focal ^18^F-FDG uptake duodenal lesions

**DOI:** 10.1097/MD.0000000000012521

**Published:** 2018-09-21

**Authors:** Ri Sa, Hong-Guang Zhao, Yu-Yin Dai, Feng Guan

**Affiliations:** Department of Nuclear Medicine, The First Hospital of Jilin University, Changchun, Jilin, China.

**Keywords:** ^18^F-FDG, dual time point, duodenal lesion, PET/CT, SUV

## Abstract

The aim of this study was to evaluate the diagnostic criteria of dual time point Fluorine-18 fluorodeoxyglucose (^18^F-FDG) positron emission tomography/computed tomography (PET/CT) in differentiating malignant from benign focal hypermetabolic lesions of duodenum.

A total of 50 patients underwent ^18^F-FDG PET/CT at 2 points: 60 ± 13.7 minutes (early imaging) and 120 ± 26.4 minutes (delayed imaging) after tracer injection. Early maximum standardized uptake value (SUVE), delayed maximum standardized uptake value (SUVD), difference between early and delayed maximum standardized uptake value (D-SUV_max_), and retention index (RI) were calculated for each duodenal lesion. Receiver operating characteristic analysis (ROC) was performed to evaluate the discriminating validity of the parameters.

There were 32 malignant and 18 benign focal ^18^F-FDG uptake duodenal lesions. The values of SUVE, SUVD, and D-SUV_max_ were significantly different between malignant and benign lesions (12.5 ± 6.3 vs 5.8 ± 1.2, 13.5 ± 6.5 vs 5.5 ± 1.1 and 0.3 ± 0.8 vs 1.0 ± 1.0, respectively). The areas under the curves (AUCs) of SUVE, SUVD, D-SUV_max_ were 0.932, 0.964 and 0.707, respectively. There was no significantly difference between SUVE and SUVD based on AUC. In detecting malignant lesions, SUVE=6.9 yielded a sensitivity of 88.9% and specificity of 84.4%, SUVD=7.2 yielded a sensitivity of 94.6% and specificity of 90.6%, D-SUV_max_=0.5 yielded a sensitivity of 72.2% and specificity of 68.8%. SUVD was the best diagnostic indicator, regarding specificity and specificity.

SUVE and SUVD had good sensitivity, specificity for differentiating duodenal lesions. But there was no significantly difference between diagnostic value of SUVE and SUVD. ^18^F-FDG uptake patterns are helpful for differentiating benign and malignant duodenal lesions.

## 1 Introduction

Small bowel tumors is rare, accounting for <2% of digestive tract tumors and the incidence of duodenal malignant tumors accounted for 33% to 45% of all small intestine tumors.^[1]^ Duodenal lesions are generally detected incidentally. The symptoms are not typical at the start. Because of the complexity of anatomic characteristics of duodenum, duodenal lesions are uncommonly found on upper endoscopy.^[2]^ Fluorine-18 fluorodeoxyglucose (^18^F-FDG) positron emission tomography/ computed tomography (PET/CT) has been shown to be an on invasive imaging modality for distinguishing benign from malignant gastrointestinal lesions. However, in the field of duodenal lesions, both benign as well as malignant duodenal lesions frequently have high ^18^F-FDG uptake. It lacks certain diagnostic value of ^18^F-FDG PET/CT to classify duodenal lesions as benign or malignant, especially it presents as a nodule or mass. The objective of this study was to investigate the role of dual time point ^18^F-FDG PET/CT in the differentiation of malignancy from benignity in incidentally detected focal ^18^F-FDG uptake duodenal lesions (nodule or mass).

## 2 Materials and methods

### 2.1 Patients

A total of 50 patients (36 males, 14 females, mean age: 59.4 ± 12.1 years, range: 28–82 years old) with focal ^18^F-FDG uptake in duodenum were referred to our institution from October 2008 to April 2017. Patients enrolled in this study met the following inclusion criteria: All lesions on PET/CT show ^18^F-FDG uptake. The definitive pathologic diagnosis of malignant duodenal lesions was confirmed by surgery or endoscopy and benign duodenal lesions was confirmed by surgery, endoscopy, follow-up information. Surgery and endoscopy were performed within 1 month after ^18^F-FDG PET/CT examination. No patients have received chemotherapy or radiation therapy before ^18^F-FDG PET/CT examination.

### 2.2 ^18^F-FDG PET/CT imaging

All patients were required to fast for at least 6 hours. Blood serum glucose level was <140 mg/dL before injection of 4.07 MBq/kg of ^18^F-FDG. Scans were performed using a hybrid PET/CT scanner (Biograph 16HR; Siemens). The scan was performed twice: an early whole-body (from the mid-thigh to the base of the skull) 60 ± 13.7 minutes after the injection^18^F-FDG, followed by a delayed scan at 120 ± 26.4 minutes (upper abdomen). PET data were acquired in 2- or 3-dimensional mode and for at least 3 minutes per bed position. A low-dose CT (120 kV, 150 mA, 0.8 seconds per CT rotation and 3.75 mm slice thickness) scan was obtained for attenuation correction. Patients were allowed to breathe normally during PET/CT acquisitions.

The images from all patients with duodenal lesions were reviewed by 2 experienced physicians in PET/CT who were blinded to the results of pathology and follow-up. Disagreements were settled by consensus. Visual and semiquantitative methods were used to summarize and analyze the PET/CT data. Maximum standardized uptake value (SUV_max_) was calculated by drawing regions of interest (ROI) around lesion suspected on attenuation-corrected PET images. The criterion for positive lesions was focally increased ^18^F-FDG uptake on duodenum that could be distinguished from background duodenum.

### 2.3 Statistics

Pathological findings were the standards of reference. SPSS version 22.0 software (IBM) was used for statistical analysis. The SUV_max_ was expressed as mean ± SD. Early maximum standardized uptake value (SUVE), delayed maximum standardized uptake value (SUVD), difference between early and delayed SUV_max_ (D-SUV_max_) and retention index (RI) between benign and malignant lesions were examined using the Mann–Whitney *U* test. To examine the applicability of SUVE, SUVD, D-SUV_max_ and RI for discrimination between benign and malignant lesions, receiver-operating-characteristic (ROC) curves were derived and the areas under the curves (AUCs) were compared. Optimal cut-off values were determined, and the sensitivity, specificity of each parameter was derived. *P* values < .05 were considered statistically significant. 
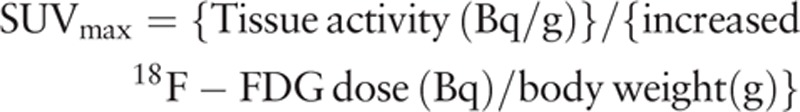
 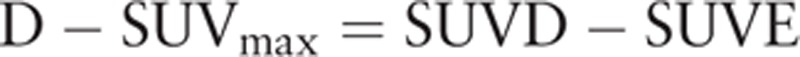 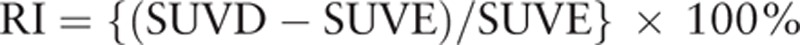


## 3 Results

### 3.1 Pathology

There were 18 benign (6 duodenal adenoma, 12 chronic inflammation of duodenal mucosa) and 32 malignant (1 diffuse large B cell lymphoma, 1 follicular lymphoma, 16 moderately differentiated adenocarcinoma, 14 well-differentiated adenocarcinoma) lesions. 11 benign and 26 malignant lesions were proven by histological examinations of surgical specimens. The other 7 benign and 6 malignant lesions were proven by histological examinations of endoscopy.

### 3.2 Sites

In 32 malignant lesions, 10 were in descending part, 10 were in descending-horizontal part, 12 were in horizontal part. In 18 benign lesions, 4 were in superior part, 4 were in descending part, 6 were in descending-horizontal part, 4 were in horizontal part.

### 3.3 Pattern

On early stage, strip distribution of ^18^F-FDG uptake occurred more frequently in benign lesions (12 patients, 66.7%) than in malignant lesions (2 patients, 6.3%), *P < *.05. Nodular distribution of ^18^F-FDG uptake was observed in 6 benign lesions (33.3%) and 30 malignant lesions (93.7%), *P < *.05.

On delayed stage: strip distribution in 12 benign lesions and nodular distribution in 2 benign lesions in early stage diffused in delayed stage. Around 4 benign lesions with nodular distribution in early stage still showed nodular distribution, but the range of lesions was smaller than earlier stage. Nodular distribution in 30 malignant lesions and strip distribution in 2 malignant lesions in early stage showed similar performance in delayed stage.

### 3.4 SUV_max_

Table 1 shows the SUV_max_ in benign and malignant focal duodenal lesions. SUVE and SUVD in malignant lesions were higher than SUVE and SUVD in benign lesions, respectively (both *P < *.001). D-SUV_max_ in malignant lesions was higher than D-SUV_max_ in benign lesions (*P < *.05). There was no significant difference in RI between benign and malignant lesions (*P > *.05).

**Table 1:**
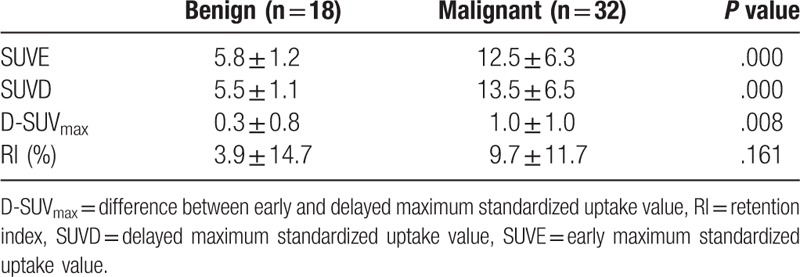
SUV_max_ in benign and malignant focal duodenal lesions.

**Figure 1:**
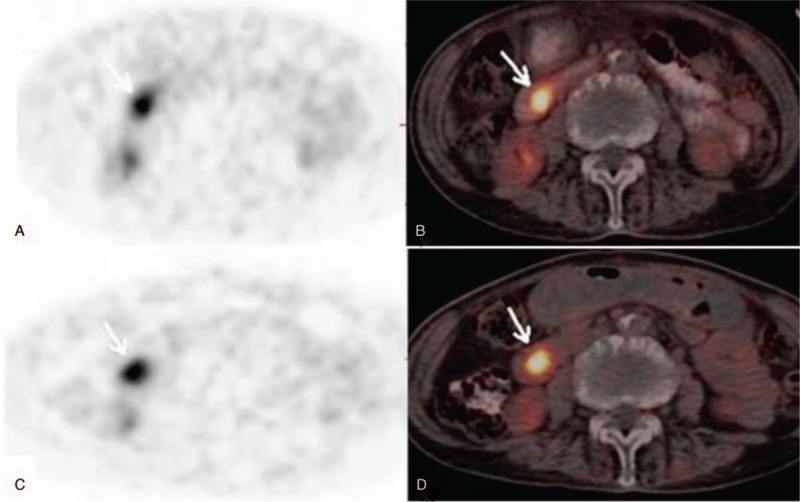
A malignant duodenal lesion (adenocarcinoma) in a 72-year-old female shows significant uptake on early (A, B) and delayed (C, D) ^18^F-FDG PET/CT imaging. The SUV_max_ increases from 7.1 on the early stage to 7.3 on the delayed stage. ^18^F-FDG = fluorine-18 fluorodeoxyglucose, PET/CT = positron emission tomography/ computed tomography, SUV_max_ = maximum standardized uptake value.

**Figure 2:**
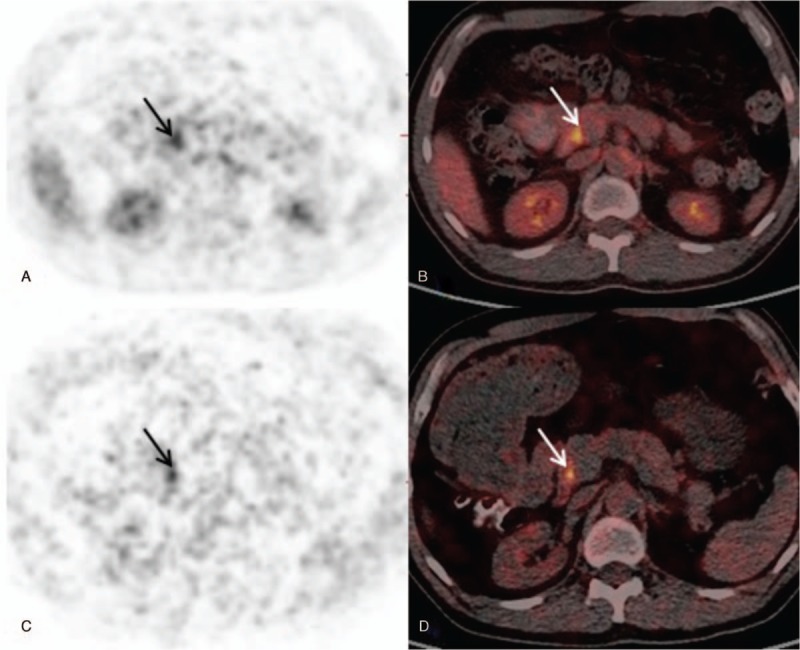
A benign duodenal lesion (adenoma) in a 45-year-old male shows ^18^F-FDG uptake on early (A, B) and delayed (C, D) PET/CT imaging. The SUV_max_ has a slight decrease from 5.2 on the early scan to 5.1 on the delayed scan. The range of delayed stage was smaller than earlier stage. ^18^F-FDG = fluorine-18 fluorodeoxyglucose, PET/CT = positron emission tomography/ computed tomography, SUV_max_ = maximum standardized uptake value.

**Figure 3:**
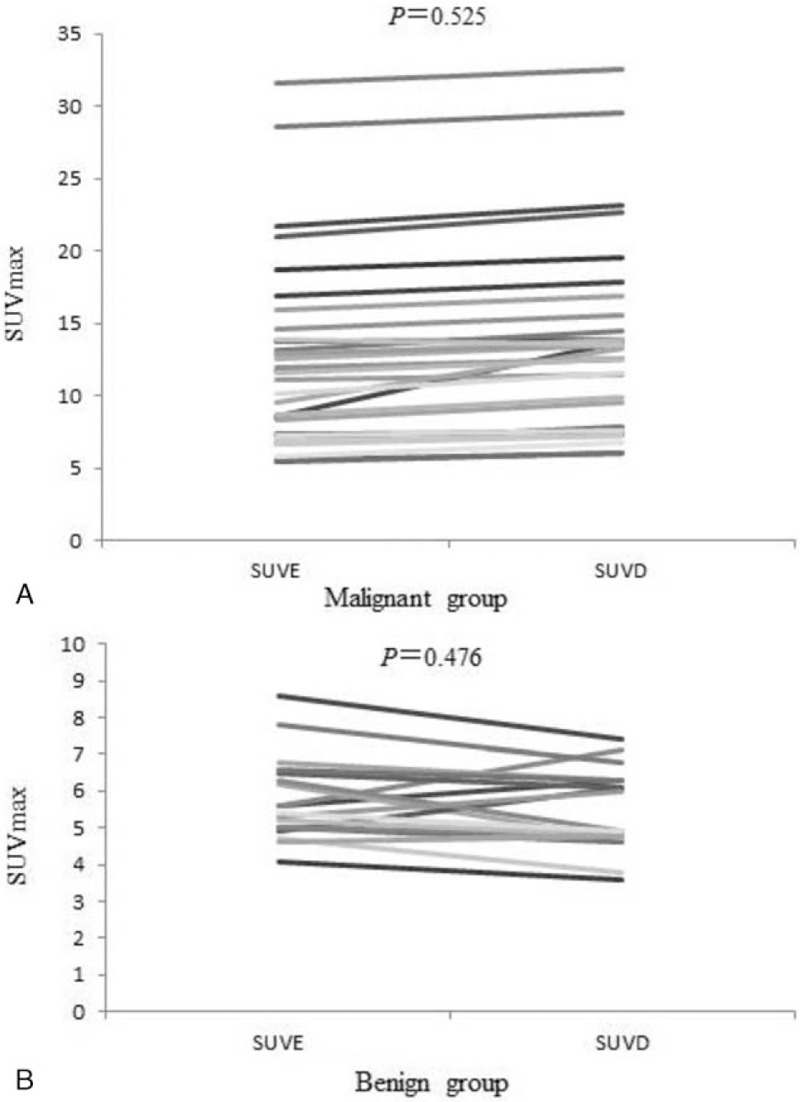
Comparison of SUVE and SUVD in malignant and benign duodenal lesions. (A) There was no statistical difference in SUVE (12.5 ± 6.3) and SUVD (13.5 ± 6.5) (*P* = .525) in malignant duodenal lesions. (B) There was no statistical difference in SUVE (5.8 ± 1.2) and SUVD (5.5 ± 1.1) (*P* = .476) in benign duodenal lesions. SUVD = delayed maximum standardized uptake value, SUVE = early maximum standardized uptake value.

In 31 malignant lesions, SUVD was higher than SUVE (RI > 0), only 1 patient had lower SUVD than SUVE (Fig. 1). In the group of benign lesions, 1 had no change in SUV_max_ (RI = 0), 5 showed higher SUVD than SUVE (RI>0) and 12 showed lower SUVD than SUVE (RI<0) (Fig. 2). However, there was no significant difference in SUVD and SUVE of malignant and benign lesions (both *P > *.05) (Fig. 3A and B).

We generated ROC curves to assess the potential usefulness of SUVE, SUVD, and D-SUV_max_ for the differentiation of malignant and benign lesions. The AUC values from the ROC curves of SUVE were 0.932 (95% confidence interval [CI] = 0.867–0.998), SUVD was 0.964 (95% CI = 0.920–1. 000), D-SUV_max_ was 0.707 (95% CI = 0.549–0.864), and RI was 0.519 (95% CI = 0.336–0.702). The optimal cut-off value of SUVE, SUVD, and D-SUV_max_ were determined by ROC curve analysis to predict the risk for malignant lesions on ^18^F-FDG PET/CT, the cut-off threshold, sensitivity, and specificity were shown in Table 2. There was no difference in AUC of SUVE and SUVD (*P > *.05).

**Table 2:**
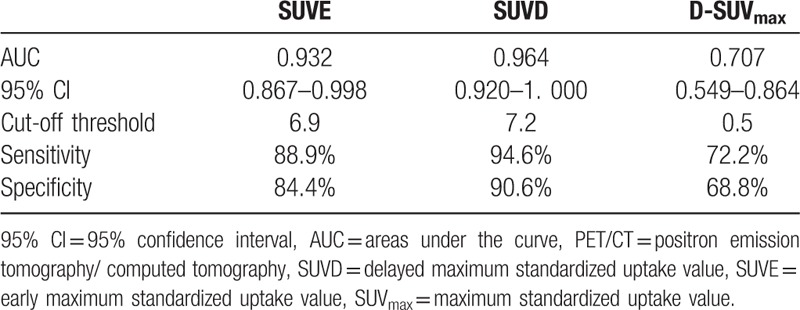
Efficacy of dual time point PET/CT in ^18^F-FDG uptake duodenal lesions.

## 4 Discussion

^18^F-FDG PET/CT is uniquely suited for evaluating metabolic activity in human tumors for diagnostic imaging purpose. The purpose of this study is to investigate the clinical role of dual time point ^18^F-FDG PET/CT for the differentiation of malignancy and benignity in incidentally detected ^18^F-FDG-uptake duodenal lesions by semiquantitative analysis the indices of dual time point PET/CT.

Once a focal duodenal lesion has been detected, its characteristics are critical to treatment plan. The differential diagnosis of duodenal lesions includes adenoma, chronic mucosal inflammation, tuberculosis, duodenal adenocarcinoma, lymphoma, neuroendocrine tumor, etc.^[3–6]^ The pathological types of ^18^F-FDG-uptake duodenal lesions included in our study were duodenal adenoma (6 cases), chronic inflammation of duodenal mucosa (12 cases), malignant lymphoma (2 cases), and adenocarcinoma (30 cases). The most common clinical conundrum is to differentiate malignant lesions from chronic inflammation of duodenal mucosa lesions.

The duodenum is a 25 to 38 cm C-shaped structure lying adjacent to the stomach. It is divided into 4 parts: superior part, descending part, horizontal part, and ascending part. Involvement of any region of the duodenum is possible. In our study, the most commonly affected parts in malignant lesions were descending and horizontal parts, while in benign lesions, the involvement sites were superior part, descending part and horizontal part.

In this study, ^18^F-FDG uptake patterns were classified as strip and nodular distribution. The 2 patterns were differentially observed in benign and malignant patients. The strip distribution was an indicator of benign lesions. 66.7% benign lesions showed strip distribution while only 6.3% malignant lesions showed strip distribution on both early and delayed stages. The nodular distribution was a significant indicator of malignant lesions, up to 93.7% patients. 4 benign lesions showed nodular distribution on both early and delayed stages and finally pathology confirmed as duodenal adenoma. With regard to ^18^F-FDG uptake pattern, duodenal adenoma has the similar performance with malignant lesions on early stage, but the range of duodenal adenoma in delayed stage was smaller than earlier stage.

In many malignant tumors, SUV does not reach maximum levels until several hours after ^18^F-FDG injection, so scan start times of 45 to 60 minutes have been reported to cause significant underestimation of the true SUV.^[7]^^18^F-FDG uptake lesions were measured in ROIs drawn on the PET image and SUV_max_ was measured. The SUV measured on ^18^F-FDG PET/CT is a semiquantitative measure of the degree of glucose uptake in lesions.^[8]^ According to previous studies of dual time point ^18^F-FDG PET/CT, the SUVD is significantly higher than SUVE in many malignant tumors, such as solitary pulmonary lesions, malignant lymphoma and gastrointestinal cancers, while benign lesions have a tendency to decrease or subtle increase of SUV_max_.^[9–12]^ In the study of Miyake et al,^[11]^ 8 focal ^18^F-FDG-uptake in the small intestine were found on the early scan (1-h postinjection) and 5 (63%) and 8 (100%) lesions were accurately interpreted as physiologic by considering changes in the appearance with time at 85 ± 7 minutes and 124 ± 7 minutes postinjection, respectively. But dual time point imaging did not always work in differentiation between benign and malignant lesions.^[13]^ Choi et al reported that dual time point imaging did not improve the overall performance of ^18^F-FDG PET/CT in detecting axillary lymph node metastasis in breast cancer patients.^[14,15]^ In principle, RI is a useful index because it has a merit of not being dependent on scale.^[7]^ In our study, we analyses SUV_max_ of early and delayed phase (SUVE, SUVD), D-SUV_max_ and RI. SUVE, SUVD and D-SUV_max_ of the malignant duodenal lesions were significantly higher than those of benign duodenal lesions while there was no significant difference in RI between the 2 groups. These results are consistent with that of Nakayama et al.^[10]^ The reason why RI was not the best predictor is still unknown.

SUVE, SUVD and D-SUV_max_ are useful predictors of malignancy. Our ROC analyses revealed that the AUC of SUVD was the greatest among these 4 indices, with 94.6 % sensitivity and 90.0 % specificity at a SUVD cut-off level of 7.2, while we found sensitivity and specificity values of 88.9 % and 84.4% using SUVE cut-off level 6.9. Hence, when SUV_max_ is used for differential diagnosis of incidentally detected ^18^F-FDG uptake duodenal lesion, there is a chance of making the diagnostic decision.

This study had some limitations. First, the population of the study was small (n = 50). Only several pathological types of duodenal lesions were involved in our study and adenocarcinoma accounted for roughly 60% of all patients. Therefore, the findings of our study may reflect the PET/CT characteristics of adenocarcinoma. We still need larger scaled studies to represent the differentiation ability of ^18^F-FDG uptake duodenal lesions. Second, this study was quantitative. It can be argued whether quantitative or qualitative analysis is more effective in distinguishing between malignant and benign duodenal lesions.

In conclusion, this study assessed the usefulness of early and delayed SUV_max_ (SUVE, SUVD), D-SUV_max_ and RI in duodenal ^18^F-FDG uptake lesions. We found that SUVE and SUVD had good sensitivity, specificity for differentiating duodenal lesions. But there was no significantly difference between diagnostic value of SUVE and SUVD. In addition, ^18^F-FDG uptake patterns are helpful for differentiating benign and malignant duodenal lesions.

## Author contributions

**Conceptualization:** Feng Guan.

**Data curation:** Ri Sa, Yu-Yin Dai, Feng Guan.

**Formal analysis:** Ri Sa, Feng Guan.

**Investigation:** Feng Guan.

**Methodology:** Feng Guan.

**Project administration:** Feng Guan.

**Resources:** Yu-Yin Dai.

**Software:** Hong-Guang Zhao.

**Supervision:** Hong-Guang Zhao, Feng Guan.

**Validation:** Hong-Guang Zhao.

**Visualization:** Hong-Guang Zhao, Feng Guan.

**Writing – original draft:** Ri Sa, Feng Guan.

**Writing – review & editing:** Ri Sa, Feng Guan.
